# The influence of methotrexate-related transporter and metabolizing enzyme gene polymorphisms on peri-engraftment syndrome and graft-versus-host disease after haplo-hematopoietic stem cell transplantation in pediatric patients with malignant hematological diseases

**DOI:** 10.3389/fimmu.2023.1229266

**Published:** 2023-09-05

**Authors:** Qi Ji, Yongping Zhang, Yixin Hu, Lixia Liu, Shanbo Cao, Li Gao, Bohan Li, Yuanyuan Tian, Lingjun Kong, Shuiyan Wu, Jing Ling, Peifang Xiao, Jun Lu, Jie Li, Yanhua Yao, Jiayue Qin, Shaoyan Hu

**Affiliations:** ^1^ Department of Hematology and Oncology, Children’s Hospital of Soochow University, Suzhou, China; ^2^ Department of Medical Affairs, Acornmed Biotechnology Co., Ltd., Tianjin, China; ^3^ Pediatric Intensive Care Unit, Children’s Hospital of Soochow University, Suzhou, China

**Keywords:** MTX, Peri-ES, GvHD, *SLCO1B1*, gene polymorphism

## Abstract

**Background:**

Methotrexate (MTX), utilized as a graft-versus-host disease (GvHD) prophylactic agent in allogeneic hematopoietic stem cell transplantation (allo-HSCT), has been proven to effectively decrease the occurrence of the peri-engraftment syndrome (Peri-ES) and acute GvHD (aGvHD). Changes in the pharmacodynamics of MTX are closely associated with gene polymorphisms in genes encoding drug-metabolizing enzymes and transporters. Nevertheless, the current studies mainly concentrate on leukemia or autoimmune diseases, and limited studies on allo-HSCT were reported.

**Methods:**

Here, we retrospectively assessed the relationship between MTX-related transporter and metabolizing enzyme gene polymorphisms, clinical characteristics, and outcomes in 57 pediatric patients who received haploid HSCT (haplo-HSCT) with malignant tumors at a single center.

**Results:**

We discovered all gene polymorphisms were in the Hardy–Weinberg equilibrium in our cohort. We discovered a significant correlation between platelet recovery time and *ABCB1* (1236C>T) (*p* = 0.042). Compared with patients with *SLCO1B1* (1865+4846T>C) TT, patients with *SLCO1B1* (1865+4846T>C) TC/CC had an increased incidence of Peri-ES (*p* = 0.030). Based on the multivariate Cox analysis, we discovered that *SLCO1B1* (1865+4846T>C) TT genotype was an independent protective factor for Peri-ES morbidity (hazard ratio (HR) = 0.464, *p* = 0.031), and the dose of mononuclear cells reinfused was significantly correlated with II–IV aGvHD (HR = 2.604, *p* = 0.039).

**Conclusion:**

In summary, our findings prove that the host’s genotypes might modify the risk of developing Peri-ES, contribute to a better understanding of the inter-individual difference in efficacy, and facilitate the development of individualized approaches to GvHD prophylaxis.

## 1 Introduction

Allogeneic hematopoietic stem cell transplantation (allo-HSCT) is an effective method to treat hematological malignancies. However, patients still face various complications after transplantation, including peri-engraftment syndrome (Peri-ES), graft-versus-host disease (GvHD), and infection, which adversely affect the survival and prognosis of patients (1–3). Methotrexate (MTX) is one of the main drugs used to prevent GvHD after HSCT, which effectively reduces the incidence of Peri-ES and acute GvHD (aGvHD) (4). However, MTX may also cause various adverse reactions, including severe mucositis, bone marrow suppression, hepatotoxicity, etc. (4, 5). Changes in the pharmacodynamics of MTX are closely associated with gene polymorphisms in genes encoding drug-metabolizing enzymes and transporters, as demonstrated by the therapy of acute lymphocytic leukemia with high-dose MTX (HD-MTX), which were rarely reported in populations undergoing allo-HSCT. Limited reports had focused on the association between gene polymorphisms and Peri-ES.

This study aims to verify the effects of MTX-related transporter and metabolizing enzyme gene polymorphisms on Peri-ES and aGvHD after haploid HSCT (haplo-HSCT) in children.

## 2 Materials and methods

### 2.1 Study design and subjects

This retrospective longitudinal cohort study was conducted in a single center at the Children’s Hospital of Soochow University (Suzhou, China). Patients who received allo-HSCT were enrolled between January 1, 2019, and June 30, 2021. The study inclusion criteria consisted of 1) first-time transplant, 2) haplo-HSCT, 3) patients aged less than 18 years, 4) three or four intravenous doses of MTX (three doses: day 1 at 15 mg/m^2^ and days 3 and 6 at 10 mg/m^2^; four doses: day 1 at 15 mg/m^2^ and days 3, 6, and 11 at 10 mg/m^2^) and cyclosporine A or tacrolimus (po q12h starting on the day 5 before transplantation) for GvHD prophylaxis, and 5) presence of informed consent to methotrexate genotyping. Patients who had undergone a second or subsequent transplantation or received GvHD prophylaxis without MTX were excluded from the study. All available follow-up data until December 31, 2022, were analyzed.

### 2.2 Endpoint definitions

Neutrophil engraftment was defined as a neutrophil count >0.5 × 10^9^/L for three consecutive days. Platelet engraftment was defined as a platelet count exceeding 20 × 10^9^/L for seven consecutive days without transfusion. Primary graft failure (GF) was defined as the failure of neutrophil engraftment at day +28 after HSCT, while secondary GF was defined as the development of neutrophil count <0.5 × 10^9^/L occurring after the initial engraftment. Peri-ES was defined according to the previously described criteria (6). Moreover, diagnosis and grading of aGvHD were performed based on the standard criteria (7). Patients who survived >30 days post-HSCT with successful engraftment were evaluated for aGvHD analysis.

### 2.3 DNA extraction and genotyping

Genomic DNA was isolated from bone marrow at diagnosis. A targeted 27-gene sequencing panel was used for next-generation sequencing (NGS) at Acornmed Biotechnology Co., Ltd (Tianjin, China). Multiplex libraries were sequenced with the NovaSeq instrument (Illumina). The following criteria were performed to filter raw variants: average sequencing depth on target per sample ≥1,000×, mapping quality ≥30, and base quality ≥30. Alignment of the trimmed reads was performed with the Burrows–Wheeler alignment (BWA; version 0.7.12). PCR duplicates were marked with the MarkDuplicates tool from Picard. BaseRecalibrator from Genome Analysis Toolkit (GATK; version 3.8) was used to realign and recalibrate the BWA data. Variant calling was used in Mutect2. ANNOVAR software was performed to annotate all the variants, including dbSNP. The MTX-related transporter and metabolizing enzyme gene polymorphisms include *SLCO1B1* (1865+4846T>C), *SLCO1B1* (521T>C), *MTRR* (66A>G), *MTHFR* (665C>T), *ABCB1* (1236C>T), *ABCB1* (3435C>T), *ABCB1* (1000-44C>T), *ABCB1* (1554 + 24A>G), and *ABCB1* (1725 + 38C>T).

### 2.4 Statistical analysis

This primary purpose of interest was the development of Peri-ES and aGvHD after haplo-HSCT with different MTX-related transporter and metabolizing enzyme gene polymorphism status. The *t*-test and Mann–Whitney U test were routinely used for the comparison of the quantitative variables between two groups with parametric and non-parametric distribution, respectively. Categorized variables were compared using chi-square or Fisher’s exact test. Event time distributions for the clinical outcomes were estimated using a cumulative incidence curve with a log-rank test. Univariate and multivariate analyses were used to compare the relative risk by Cox regression. Variables were selected by univariate Cox regression analysis, and those with *p* < 0.2 were subsequently enrolled in multivariate Cox regression analysis based on the likelihood ratio test. All data were analyzed using SPSS software (version 22.0) or R (version 3.5.2). *p* < 0.05 was considered statistically significant.

## 3 Results

### 3.1 Demographic data and transplant-associated patient data

The baseline characteristics of 57 Chinese pediatric patients are described in **Table 1**. All 57 patients were treated with the myeloablative conditioning regimen based on busulfan (Bu) and cyclophosphamide (Cy) and received at least three doses of MTX as GvHD prophylaxis. The mean age at HSCT was 5.1 years, and more men (80.8%) were enrolled in our cohort. Acute leukemias, including mixed-phenotype acute leukemia (MAPL), was the most common etiology, which constituted 94.7% (54/57) of the cohort. All patients achieved hematopoietic recovery from haploidentical donors. The median time to neutrophil and platelet engraftment was 12 days (range, 10–23) and 12 days (range, 5–39), respectively.

**Table 1:** Baseline characteristics.

Demographic information	Patient cohort (n = 57)
Male, n (%)	46 (80.8%)
Age, median (range) months	61.267 (1.0, 195.6)
Weight, median (range) kg	28.7 (9.3, 96.5)
Diagnosis, n (%)	
AML	22 (39.2%)
ALL	30 (52.6%)
MPAL	2 (3.6%)
JMML	1 (1.8%)
CML	1 (1.8%)
T-LBL	1 (1.8%)
BM state before transplantation, n (%)	
CR1	36 (63.2%)
CR2	17 (29.8%)
PR/NR	4 (7.0%)
GvHD prophylaxis, n (%)	
MTX + CsA + others	47 (82.4%)
MTX + FK506 + others	10 (17.6%)
Transplantation, n (%)	
BM + PB + CB	29 (50.8%)
PB	12 (21.0%)
BM + PB	14 (24.6%)
BM + CB	1 (1.8%)
PB + CB	1 (1.8%)
Haplo HLA match, n (%)	
5/10	34 (59.6%)
6/10–8/10	19 (33.4%)
9/10–10/10	4 (7.0%)
MNC, median (range) ×10^8^/kg	6.99 (1.56, 21.71)
CD34 cell, median (range) ×10^6^/kg	7.17 (1.06, 22.4)
Neutrophil recovery day, median (range) day	12 (10, 23)
Platelet recovery day, median (range) day	12 (5, 39)
Donor–patient sex match, n (%)	
Female–male	7 (12.2%)
Male–male	39 (68.4%)
Female–female	4 (7.0%)
Male–male	7 (12.2%)

AML, acute myeloid leukemia; ALL, acute lymphoblastic leukemia; MAPL, mixed-phenotype acute leukemia; JMML, juvenile myelomonocytic leukemia; CML, chronic myeloid leukemia; T-LBL, T-cell lymphoblastic lymphoma; BM, bone marrow; CR, complete remission; PR, partial remission; NR, no remission; GvHD, graft-versus-host disease; MTX, methotrexate; CsA, cyclosporine A; FK506, tacrolimus; PB, peripheral blood; CB, cord blood; MNC, mononuclear cells.

CR1 means that after chemotherapy and other treatments, the BM has achieved CR for the first time; CR2 means that after achieving CR for the first time, the patient experiences relapse and achieves CR again through further treatments.

### 3.2 MTX-related transporter and metabolizing enzyme gene polymorphism frequencies

**Figure 1:**
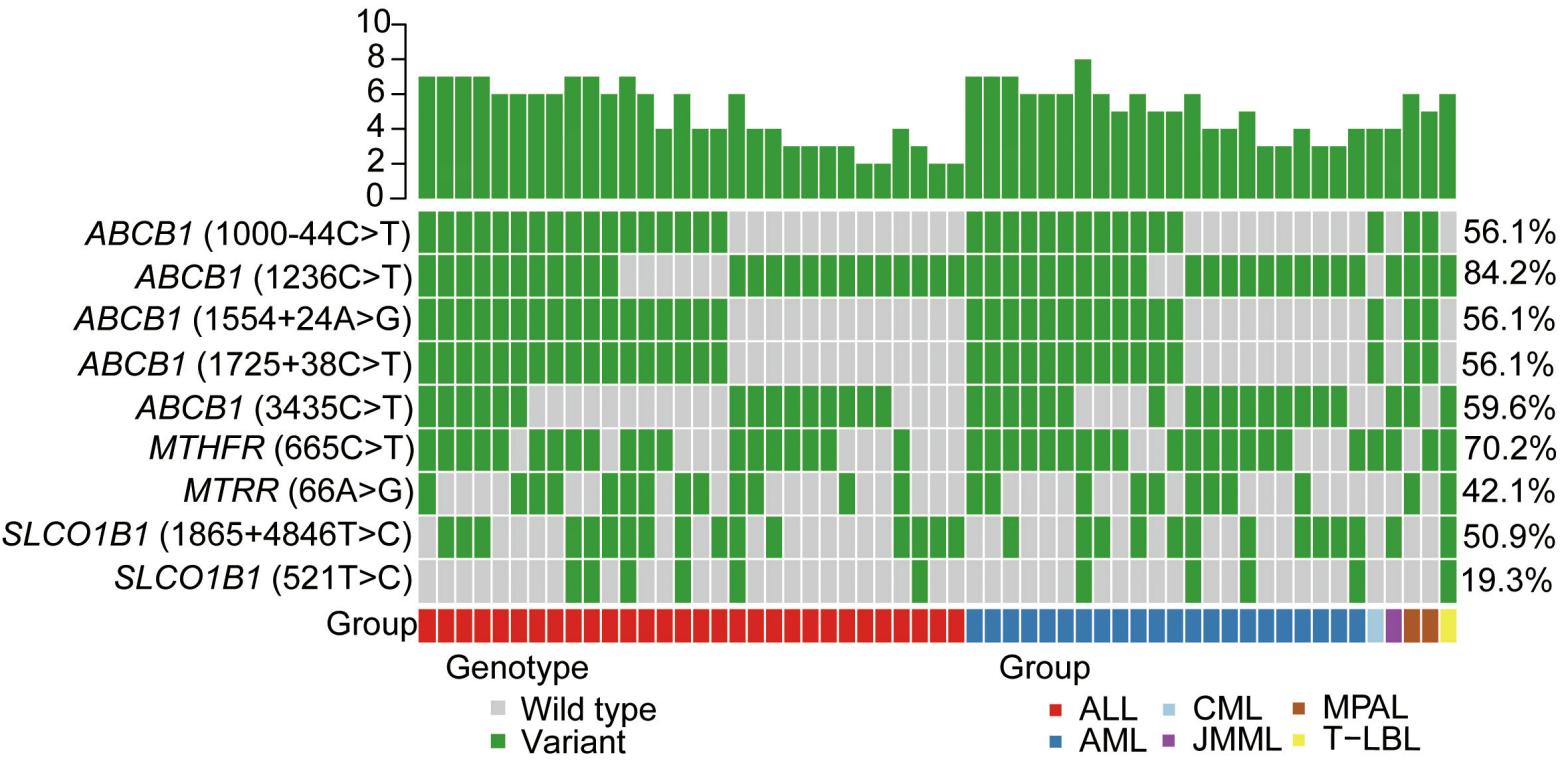
MTX-related transporter and metabolizing enzyme gene polymorphisms detected in our cohort. Heatmap shows the specific variants in each patient based on different genotypes, according to different subtypes of malignant hematological diseases. MTX, methotrexate; ALL, acute lymphoblastic leukemia; AML, acute myeloid leukemia; CML, chronic myeloid leukemia; JMML, juvenile myelomonocytic leukemia; MPAL, mixed-phenotype acute leukemia; T-LBL, T-cell lymphoblastic lymphoma.

MTX-related transporter and metabolizing enzyme gene polymorphism frequencies for the patients are shown in **Figure 1** and **Table 2**. A total of nine kinds of gene polymorphisms were detected, and the most common variant type was *ABCB1* (1236C>T), followed by *MTHFR* (665C>T) and *ABCB1* (3435C>T) (**Figure 1**). All MTX-related transporter and metabolizing enzyme gene polymorphisms were discovered to be in the Hardy–Weinberg equilibrium in our cohort (all *p* > 0.05, **Table 2**). In addition, *ABCB1* (1236C>T) was found to be related to platelet recovery day (*p* = 0.042, **Supplementary Table 1**).

**Table 2:** MTX-related transporter and metabolizing enzyme gene polymorphism frequencies.

Gene	SNP	Genotype	Patient, n (%)	Theoretical frequency, n	*p*-Value
*SLCO1B1*	*SLCO1B1* (1865+4846T>C), rs11045879	TT	28 (49.1%)	28.070	0.965
		TC	24 (42.1%)	23.860	
		CC	5 (8.8%)	5.070	
	*SLCO1B1* (521T>C), rs4149056	TT	46 (80.7%)	45.632	0.604
		TC	10 (17.5%)	10.737	
		CC	1 (1.8%)	0.632	
*MTRR*	*MTRR* (66A>G), rs1801394	AA	33 (57.9%)	33.965	0.469
		AG	22 (38.6%)	20.070	
		GG	2 (3.5%)	2.965	
*MTHFR*	*MTHFR* (665C>T), rs1801133	CC	17 (29.8%)	15.789	0.520
		TC	26 (45.6%)	28.421	
		TT	14 (24.6%)	12.789	
*ABCB1*	*ABCB1* (1236C>T), rs1128503	CC	9 (15.7%)	7.373	0.349
		TC	23 (40.4%)	26.254	
		TT	25 (43.9%)	23.373	
	*ABCB1* (3435C>T), rs1045642	CC	23 (40.4%)	21.491	0.399
		TC	24 (42.1%)	27.018	
		TT	10 (17.5%)	8.491	
	*ABCB1* (1000-44C>T), rs10276036	CC	25 (43.9%)	23.373	0.349
		TC	23 (40.3%)	26.254	
		TT	9 (15.7%)	7.373	
	*ABCB1* (1554+24A>G), rs2235033	AA	25 (43.9%)	22.110	0.103
		AG	21 (36.8%)	26.781	
		GG	11 (19.3%)	8.110	
	*ABCB1* (1725+38C>T), rs2235013	CC	25 (43.9%)	22.110	0.103
		TC	21 (36.8%)	26.781	
		TT	11 (19.3%)	8.110	

MTX, methotrexate; SNP, single-nucleotide polymorphism.

### 3.3 Frequency and time to onset of Peri-ES

**Figure 2:**
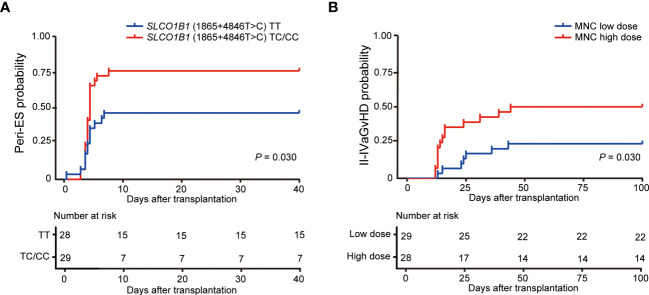
Effects of *SLCO1B1* (1865+4846T>C) gene polymorphisms and MNC doses on the morbidity of Peri-ES **(A)** and II–IV aGvHD **(B)**, respectively. Peri-ES, Peri-engraftment syndrome; aGvHD, acute graft-versus-host disease; MNC, mononuclear cells.

In this study, 61.4% of patients (35/57) developed Peri-ES; 94% of patients (33/35) with Peri-ES received methylprednisolone therapy and improved. There was an increased morbidity rate of Peri-ES in the patients with *SLCO1B1* (1865+4846T>C) TC/CC (*p* = 0.030, **Figure 2A**). Based on multivariate analysis, *SLCO1B1* (1865+4846T>C) TT genotype (hazard ratio (HR) = 0.464, *p* = 0.031) was an independent protective factor for Peri-ES morbidity. However, no association was found between other gene polymorphisms with Peri-ES, such as *ABCB1*, *MTHFR*, and *MTRR* polymorphisms (**Table 3**).

**Table 3:** Univariate and multivariate Cox regression analyses of Peri-ES.

Characteristics	Univariate analysis		Multivariate analysis	
	HR (95% CI)	*p*-Value	HR (95% CI)	*p*-Value
Female *vs.* male	0.559 (0.254–1.228)	0.189		
Age, ≤5 years *vs.* >5 years	1.504 (0.543–2.045)	0.886		
Using TBI, yes *vs.* no	0.441 (0.159–1.217)	0.213		
GvHD prophylaxis, FK506 + MTX + others *vs.* CsA + MTX + others	0.733 (0.313–1.720)	0.489		
Using CB, no *vs.* yes	0.812 (0.417–1.580)	0.515		
HLA				
6/10-8/10 *vs.* 5/10	0.523 (0.266–1.029)	0.062		
9/10-10/10 *vs.* 5/10	0.000 (0.000–Inf)	0.014		
MNC, high *vs.* low dose	1.271 (0.653–2.471)	0.446		
CD34+ cell, high *vs.* low dose	1.148 (0.591–2.228)	0.661		
*SLCO1B1* (1865+4846T>C), TT *vs.* TC/CC	0.503 (0.259–0.978)	0.030	0.464 (0.231–0.931)	0.031
*SLCO1B1* (521T>C), TT *vs.* TC/CC	0.683 (0.282–1.656)	0.305		
*MTRR* (66A>G), AA *vs.* AG/GG	0.876 (0.445–1.724)	0.677		
*MTHFR* (665C>T), CC *vs.* TC/TT	0.726 (0.358–1.475)	0.373		
*ABCB1* (1236C>T), CC *vs.* TC/TT	0.841 (0.345–2.051)	0.699		
*ABCB1* (3435C>T), CC *vs.* TC/TT	0.956 (0.485–1.887)	0.890		
*ABCB1* (1000-44C>T), CC *vs.* TC/TT	1.136 (0.581–2.219)	0.687		
*ABCB1* (1554+24A>G), AA *vs.* AG/GG	1.136 (0.581–2.219)	0.687		
*ABCB1* (1725+38C>T), CC *vs.* TC/TT	1.136 (0.581–2.219)	0.687		
Donor gender, male *vs.* female	1.485 (0.647–3.408)	0.377		

Peri-ES, peri-engraftment syndrome; HR, hazard ratio; TBI, total body irradiation; GvHD, graft-versus-host disease; MTX, methotrexate; CsA, cyclosporine A; FK506, tacrolimus; CB, cord blood; MNC, mononuclear cells.

### 3.4 Morbidity, site, and grade of aGvHD after haplo-HSCT

In the study, 36.8% of patients (21/57) developed grade II–IV aGvHD, including eight (14.0%) with grade II, eight (19.3%) with grade III, and five (8.8%) with grade IV. With respect to organ severity stage, 20 patients developed stage 2–4 cutaneous aGvHD, and eight patients developed stage 2–4 GI aGvHD. Only one child developed stage 4 liver aGvHD. Compared with mononuclear cell (MNC) low dose according to the median dose value in our cohort, patients with MNC high dose had an increased incidence of II–IV aGvHD (*p* = 0.030, **Figure 2B**). Also, we conducted univariate and multivariate Cox regression analyses for II–IV aGvHD and III–IV aGvHD. Our findings suggested that *SLCO1B1* (521T>C) genotype tended to be associated with II–IV aGvHD in univariate analysis (*p* = 0.175), and only the dose of MNC reinfused was significantly related to II–IV aGvHD as an independent risk factor in multivariate analysis (HR = 2.604, *p* = 0.039, **Table 4**). Unfortunately, no significant association between III–IV aGvHD after haplo-HSCT and MTX-related gene polymorphisms was discovered (**Supplementary Table 2**).

**Table 4:** Univariate and multivariate Cox regression analyses of the morbidity of II–IV aGvHD.

Characteristics	Univariate analysis		Multivariate analysis	
	HR (95% CI)	*p*-Value	HR (95% CI)	*p*-Value
Female *vs.* male	1.027 (0.342–3.080)	0.961		
Age, ≤5 years *vs.* >5 years	0.783 (0.332–1.847)	0.569		
Using TBI, yes *vs.* no	1.360 (0.345–5.358)	0.616		
GvHD prophylaxis, FK506 + MTX + others *vs.* CsA + MTX + others	0.419 (0.144–1.217)	0.221		
Using CB, no *vs.* yes	0.565 (0.240–1.331)	0.205		
HLA				
6/10–8/10 *vs.* 5/10	0.870 (0.358–2.115)	0.760		
9/10–10/10 *vs.* 5/10	0.000 (0.000–Inf)	0.146		
MNC, high *vs.* low dose	2.594 (1.095–6.146)	0.030	2.604 (1.049–6.463)	0.039
CD34+ cell, high *vs.* low dose	1.157 (0.492–2.723)	0.735		
*SLCO1B1* (1865+4846T>C), TT *vs.* TC/CC	1.149 (0.489–2.704)	0.747		
*SLCO1B1* (521T>C), TT *vs.* TC/CC	0.529 (0.172–1.633)	0.175		
*MTRR* (66A>G), AA *vs.* AG/GG	1.494 (0.629–3.547)	0.376		
*MTHFR* (665C>T), CC *vs.* TC/TT	0.676 (0.269–1.696)	0.436		
*ABCB1* (1236C>T), CC *vs.* TC/TT	1.220 (0.382–3.897)	0.717		
*ABCB1* (3435C>T), CC *vs.* TC/TT	0.735 (0.307–1.759)	0.473		
*ABCB1* (1000-44C>T), CC *vs.* TC/TT	1.200 (0.506–2.845)	0.672		
ABCB1 (1554+24A>G), AA *vs.* AG/GG	1.200 (0.506–2.845)	0.672		
*ABCB1* (1725+38C>T), CC *vs.* TC/TT	1.200 (0.506–2.845)	0.672		
Peri-ES, no *vs.* yes	0.556 (0.234–1.324)	0.212		
Donor gender, male *vs.* female	1.490 (0.510–4.350)	0.514		

GvHD, graft-versus-host disease; HR, hazard ratio; TBI, total body irradiation; MTX, methotrexate; CsA, cyclosporine A; FK506, tacrolimus; CB, cord blood; MNC, mononuclear cells; Peri-ES, peri-engraftment syndrome.

## 4 Discussion

In this study, we analyzed the correlation between MTX-related transporter and metabolizing enzyme gene polymorphisms, Peri-ES, and aGvHD after haplo-HSCT in 57 pediatric patients with malignant hematological diseases. Basic characteristics and transplant outcomes between different MTX-related transporter and metabolizing enzyme gene polymorphisms were compared, in which we primarily found that patients with *ABCB1* (1236C>T) were associated with platelet recovery day. When considering the time of disease occurrence, we found that patients with *SLCO1B1* (1865+4846T>C) TC/CC genotype had a higher incidence of Peri-ES. Furthermore, our Cox regression analysis revealed that *SLCO1B1* (1865+4846T>C) TT genotype was an independent protective factor against the overall incidence of Peri-ES.

Peri-ES is defined as a clinical syndrome encompassing both engraftment syndrome (ES) and pre-engraftment syndrome (pre-ES), which is closely correlated with the prognosis (6). In our cohort, 61.4% of patients (35/57) developed Peri-ES, which was higher than the reported rate (6). Our data showed that patients receiving haplo-HSCT combined with umbilical cord blood (haplo-cord HSCT) had a trend of increasing incidence of Peri-ES (21/31 *vs.* 14/26, *p* = 0.283). It is well known that the incidence of Peri-ES is closely associated with umbilical cord blood transplantation (UCBT). Previous studies showed that granulocyte-macrophage colony-stimulating factor (GM-CSF) produced by cord blood-derived inflammatory monocytes played a crucial role in driving the pathology of pre-ES in UCBT (8). In our study, approximately half of the patients underwent haplo-HSCT combined with UCBT. Thus, the increased vulnerability to Peri-ES in our cohort may be linked to the presence of highly potent monocytes that produce GM-CSF within the UCB grafts. It was noteworthy that an overwhelmingly high proportion of patients underwent methylprednisolone therapy and improved.

MTX is a cornerstone drug in preventing GvHD and has been shown to significantly reduce the incidence of Peri-ES after UCBT (9). The disposition of MTX is a complex process that involves various transporters and enzymes. Renal excretion is the primary route of elimination for MTX. Due to the significant contribution of enterohepatic circulation to its disposition, both transporters in the kidneys and liver are important determinants of clearance (10). SLCO1B1, also known as OATP-2 or LST1, is a bidirectional transporter with 12 transmembrane domains encoded by *SLCO1B1* located on chromosome 12p12, which is mainly expressed in the hepatocyte basolateral membrane and plays a crucial role in the uptake of endogenous and exogenous anionic compounds, including MTX (11).

The rs11045879 single-nucleotide polymorphism (SNP), also known as *SLCO1B1* (1865+4846T>C), is an intronic variant of the *SLCO1B1* gene located at chr12:21229685. The functional implications of *SLCO1B1* (1865+4846T>C) have not been fully elucidated; however, its clinical relevance in MTX treatment is noteworthy. *SLCO1B1* (1865+4846T>C) was identified in a genome-wide association study as one of the most important factors influencing MTX clearance in patients with acute lymphoblastic leukemia (ALL) (12). Several studies have shown that patients carrying *SLCO1B1* (1865+4846T>C) C allele had a tendency to demonstrate increased MTX plasma concentrations and decreased MTX clearance rates, which is associated with an increased risk for MTX-related toxicity, including gastrointestinal toxicity and anal mucositis (12–17). Katja Goričar conducted a prognostic analysis on osteosarcoma patients undergoing HD-MTX therapy and discovered that *SLCO1B1* (1865+4846T>C) C allele was associated with a higher event-free survival (16). The studies regarding the relationship between low-dose MTX and *SLCO1B1* gene polymorphisms were rare. İrem Eldem found that the *SLCO1B1* (1865+4846T>C) variant was associated with lower tolerances to MTX in children with ALL who had completed or were receiving maintenance therapy (18). In summary, the *SLCO1B1* (1865+4846T>C) variant is closely associated with the pharmacokinetics and pharmacodynamics of MTX and deserves further investigation.

Our study suggested that *SLCO1B1* gene polymorphism was related to Peri-ES, but the mechanism was unclear. The mechanism of MTX entering cells mainly involves the carrier molecules on the cell membrane, such as members of the classic anti-cancer drug transporter family *in vitro* and *in vivo*, which recognize and transport MTX-containing complexes on the cell membrane (19). Additionally, high concentrations of extracellular MTX can allow for passive diffusion into the cell (20, 21). As the dose of MTX used for GvHD is low, MTX is more likely to enter cells *via* transporters in children undergoing allo-HSCT. Previous studies had shown that short-term MTX was related to a lower rate of ES and III–IV aGvHD (4, 22). Narimatsu indicated that MTX may alter interactions among donor lymphocytes, facilitating engraftment and residual recipient immune cells capable of graft rejection, thereby modulating engraftment (22). However, as a folic acid antagonist, MTX requires cellular entry to exert its anti-proliferative function. Most *SLCO1B1* gene polymorphisms can lead to decreased transport activity, including influx and efflux (12, 15, 23, 24), which decrease MTX entering cells. This may explain why patients with the *SLCO1B1* variant genotype showed a higher incidence of Peri-ES than those with the wild-type genotype. Another explanation for our conclusion is that although several studies had shown that *SLCO1B1* (1865+4846T>C) variant leads to increasing plasma concentration and thus results in MTX-related toxicity, these are all focused on the use of HD-MTX (13, 25, 26). Currently, there is no research on the influence of SLC family gene polymorphism on MTX after HSCT. The pharmacokinetics of the SLC family in the allo-HSCT population remains unclear. Therefore, we plan to utilize high-performance liquid chromatography (HPLC) to detect the plasma concentration of MTX after allo-HSCT to verify our results in the future.

Another interesting finding in our research was that *ABCB1* (1236C>T) was associated with platelet recovery day. MTX is pumped out from the cells by ABC subfamily transporters, including ABCB1 (27). *ABCB1* (1236C>T) was the most common and extensively studied *ABCB1* SNP, which involves a C to T transition at position 1236 within exon 12 (28). Bo Jiang discovered that the T allele of *ABCB1* (1236C>T) gene variant increases the efflux of MTX (29). This implies that in wild-type patients, there is a decrease in the efflux of MTX in hematopoietic cells, leading to the inhibition of cell proliferation by MTX. Consequently, this may explain why wild-type patients with the *ABCB1* (1236C>T) variant are more prone to experiencing slowed recovery of hematopoietic cells.

Our study has several limitations, including the absence of information regarding the donor’s gene polymorphism status and the small sample size to analyze the association between transplantation outcome and MTX-related transporter and metabolizing enzyme gene polymorphisms. Consequently, we need future studies to validate our findings in a larger subsequent cohort, especially in a prospective study design.

## 5 Conclusion

In summary, our study suggests that *SLCO1B1* genotype is correlated with Peri-ES in pediatric patients undergoing haplo-HSCT with MTX as GvHD prophylaxis. Specifically, the *SLCO1B1* (1865+4846T>C) TT genotype was an independent protective factor against the development of Peri-ES, and the dose of reinfused MNC was significantly associated with II–IV aGvHD. Our findings contribute to a better understanding of the inter-individual difference in efficacy and facilitate the development of individualized approaches to GvHD prophylaxis, ultimately leading to improved HSCT outcomes.

## Data availability statement

The datasets presented in this study can be found in online repositories. The names of the repository/repositories and accession number(s) can be found below: CNGB Sequence Archive (CNSA) of China National GeneBank DataBase (CNGBdb) CNP0004415 (https://db.cngb.org).

## Ethics statement

The studies involving humans were approved by Children’s Hospital of Soochow University Institutional Review Board. The studies were conducted in accordance with the local legislation and institutional requirements. Written informed consent for participation in this study was provided by the participants’ legal guardians/next of kin.

## Author contributions

SH and JQ designed the study and approved the final manuscript. QJ, YZ, YH, LG, BL, YT, LK, SW, JLing, PX, JLu, JLi, and YY collected the clinical sample and data. LL and CS performed the NGS platform. QJ, YZ, YH, LL, and JQ did the statistical analysis. QJ, JQ, and SH wrote and edited the manuscript. All authors contributed to the article and approved the submitted version.
