# Supplementary material for: The influence of methotrexate-related transporter and metabolizing enzyme gene polymorphisms on peri-engraftment syndrome and graft-versus-host disease after haplo-hematopoietic stem cell transplantation in pediatric patients with malignant hematological diseases
Source: Front Immunol. 2023 Sep 5;14:1229266. doi: 10.3389/fimmu.2023.1229266 (PMC10507719; doi:10.3389/fimmu.2023.1229266)
Supplement: Supplementary file 1 [file Table_1.docx]

**Supplementary Table 1.** Comparison of engraftment according to MTX-related transporter and metabolizing enzyme gene polymorphism

| SNP | Neutrophil recovery day | *P* value | Platelet recovery day | *P* value |
| --- | --- | --- | --- | --- |
| *SLCO1B1* (1865+4846T>C) |  | 0.146 |  | 0.409 |
| TT | 11 (10, 16) |  | 11.5 (5, 39) |  |
| TC/CC | 12 (10, 23) |  | 12 (7, 24) |  |
| *MTRR* (66A>G) |  | 0.221 |  | 0.703 |
| AG/GG | 11 (10, 16) |  | 12 (8, 39) |  |
| AA | 12 (10, 23) |  | 12 (5, 24) |  |
| *MTHFR* (665C>T) |  | 0.599 |  | 0.158 |
| TC/TT | 12 (10, 23) |  | 12 (5, 22) |  |
| CC | 11 (11, 16) |  | 13 (8, 39) |  |
| *SLCO1B1* (521T>C) |  | 0.460 |  | 0.590 |
| TT | 12 (10, 23) |  | 12 (5, 39) |  |
| TC/CC | 11 (11, 16) |  | 12 (9, 19) |  |
| *ABCB1* (1236C>T) |  | 0.677 |  | 0.042 |
| CC | 12(10, 16) |  | 16(10, 24) |  |
| TC/CC | 12(10, 23) |  | 11.5(5, 39) |  |
| *ABCB1* (3435C>T) |  | 0.249 |  | 0.097 |
| TC/CC | 12(11, 23) |  | 11.5(5, 39) |  |
| CC | 11(10, 16) |  | 13(8, 24) |  |
| *ABCB1* (1000-44C>T) |  | 0.404 |  | 0.410 |
| CC | 11(10, 16) |  | 13(8, 24) |  |
| CT/TT | 12(11, 23) |  | 11.5(5, 39) |  |
| *ABCB1* (1554+24A>G) |  | 0.404 |  | 0.410 |
| AA | 11(10, 16) |  | 13(8, 24) |  |
| AG/GG | 12(11, 23) |  | 11.5(5, 39) |  |
| *ABCB1* (1725+38C>T) |  | 0.404 |  | 0.410 |
| CC | 11(10, 16) |  | 13(8, 24) |  |
| CT/TT | 12(11, 23) |  | 11.5(5, 39) |  |

Abbreviations: MTX, methotrexate; SNP, single nucleotide polymorphism.

**Supplementary Table 2.** Univariate and multivariate Cox regression analysis of the morbidity of III-IV aGvHD

| Characteristics | Univariate analysis | | Multivariate analysis | |
| --- | --- | --- | --- | --- |
|  | HR (95% CI) | *P* value | HR (95% CI) | *P* value |
| Female vs. male | 2.407 (0.537 - 10.778) | 0.137 |  |  |
| Age, ≤ 5 years vs. > 5 years | 0.641 (0.206 - 1.992) | 0.443 |  |  |
| Using TBI, yes vs. no | 1.360 (0.345 - 5.358) | 0.616 |  |  |
| GvHD prophylaxis, FK506 + MTX + others vs. CsA + MTX + others | 0.419 (0.144 - 1.217) | 0.221 |  |  |
| Using CB, no vs. yes | 0.897 (0.207 - 3.881) | 0.888 |  |  |
| HLA |  |  |  |  |
| 6/10-8/10 vs. 5/10 | 1.328 (0.405 - 4.350) | 0.626 |  |  |
| 9/10-10/10 vs. 5/10 | 0.000 (0.000 - Inf) | 0.340 |  |  |
| MNC, high vs. low dose | 2.332 (0.750 - 7.255) | 0.153 |  |  |
| CD34+ cell, high vs. low dose | 0.981 (0.316 - 3.041) | 0.973 |  |  |
| *SLCO1B1* (1865+4846T>C), TT vs. TC/CC | 2.168(0.699 - 6.728) | 0.194 |  |  |
| *SLCO1B1* (521T>C), TT vs. TC/CC | 0.660(0.152 - 2.876) | 0.529 |  |  |
| *MTRR* (66A>G) AA vs. AG/GG | 1.435 (0.456 - 4.520) | 0.552 |  |  |
| *MTHFR* (665C>T) CC vs. TC/TT | 0.415 (0.124 - 1.388) | 0.239 |  |  |
| *ABCB1* (1236C>T) CC vs. TC/TT | 0.445 (0.099 - 2.007) | 0.424 |  |  |
| *ABCB1* (3435C>T) CC vs. TC/TT | 0.664 (0.209 - 2.108) | 0.473 |  |  |
| *ABCB1* (1000-44C>T) CC vs. TC/TT | 1.866 (0.595 - 5.855) | 0.277 |  |  |
| *ABCB1* (1554+24A>G) AA vs. AG/GG | 1.866 (0.595 - 5.855) | 0.277 |  |  |
| *ABCB1* (1725+38C>T) CC vs. TC/TT | 1.866 (0.595 - 5.855) | 0.277 |  |  |
| Peri-ES, no vs. yes | 1.092 (0.343 - 3.473) | 0.880 |  |  |
| Donor gender, male vs. female | 1.230（0.297 - 5.096） | 0.788 |  |  |

Abbreviations: GvHD, graft-versus-host disease; HR, hazard ratio; TBI, total body irradiation; MTX, methotrexate; CsA, cyclosporine A; FK506, tacrolimus; CB, cord blood; MNC, mononuclear cells; Peri-ES, peri-engraftment syndrome.
